# Acute ST‐Elevation Myocardial Infarction in Patients Hospitalized for Noncardiac Conditions

**DOI:** 10.1161/JAHA.113.000004

**Published:** 2013-04-24

**Authors:** Xuming Dai, Joseph Bumgarner, Andrew Spangler, Dane Meredith, Sidney C. Smith, George A. Stouffer

**Affiliations:** 1 Division of Cardiology, University of North Carolina, Chapel Hill, NC (X.D., J.B., A.S., D.M., S.C.S., G.A.S.); 2 McAllister Heart Institute, University of North Carolina, Chapel Hill, NC (X.D., G.A.S.)

**Keywords:** myocardial infarction, reperfusion, ST elevation MI, inpatient STEMI

## Abstract

**Background:**

Major advances have been made in the treatment of ST‐elevation myocardial infarction (STEMI) in outpatients. In contrast, little is known about outcomes in STEMI that occur in patients hospitalized for a noncardiac condition.

**Methods and Results:**

This was a retrospective, single‐center study of inpatient STEMIs from January 1, 2007, to July 31, 2011. Forty‐eight cases were confirmed to be inpatient STEMIs of a total of 139 410 adult discharges. These patients were older and more often female and had higher rates of chronic kidney disease and prior cerebrovascular events compared with 227 patients with outpatient STEMIs treated during the same period. Onset of inpatient STEMI was heralded most frequently by a change in clinical status (60%) and less commonly by patient complaints (33%) or changes on telemetry. Coronary angiography and percutaneous coronary intervention were performed in 71% and 56% of patients, respectively. The median time to obtain ECG (41 [10, 600] versus 5 [2, 10] minutes; *P*<0.001), ECG to angiography time (91 [26, 209] versus 35 [25, 46] minutes; *P*<0.001) and ECG to first device activation (FDA) (129 [65, 25] versus 60 [47, 76] minutes; *P*<0.001) were longer for inpatient versus outpatient STEMI. Survival to discharge was lower for inpatient STEMI (60% versus 96%; *P*<0.001), and this difference persisted after adjusting for potential confounders.

**Conclusions:**

Patients who develop a STEMI while hospitalized for a noncardiac condition are older and more often female, have more comorbidities, have longer ECG‐to‐FDA times, and are less likely to survive than patients with an outpatient STEMI.

## Introduction

Rapid reperfusion with early percutaneous coronary intervention (PCI) is the standard of care for patients with ST‐elevation myocardial infarction (STEMI) who present to a hospital with interventional cardiology capabilities. Recognition of the importance of rapid reperfusion has led to significant improvement in door‐to‐balloon times in the last 5 years (from a median of 96 minutes in 2005 to 64 minutes in 2010), which correlates with an improvement in overall outcomes of STEMI patients.^1^ Research and nationwide initiatives on STEMI care have targeted patients who have onset of STEMI while outside the hospital. Much less is known about clinical presentation, sources of delay in diagnosis and treatment, and outcomes in patients who develop STEMIs while hospitalized for a noncardiac condition. Patients in the hospital for treatment of active medical or surgical conditions are susceptible to developing STEMIs, but we know surprisingly little about optimal care in this setting.

## Methods

This was a single‐center retrospective study performed at the University of North Carolina Hospitals (UNC), an academic tertiary health care facility. After approval by the institutional review board, we searched the hospital discharge database from January 1, 2007, to July 31, 2011, for patients with a diagnosis code of “acute myocardial infarction, not present on admission” using ICD‐9 codes 410.00 to 410.92. For comparison, we identified all outpatients with STEMI who presented directly to UNC during this period using a STEMI database maintained by the hospital (patients who were transferred from another health care facility were excluded).

History of cerebrovascular accident or transient ischemia attack was obtained from the medical record. Sleep apnea, hypertension, diabetes mellitus, and/or chronic obstructive pulmonary disease (COPD) were considered present if the patient was receiving treatment. Known coronary artery disease was present if the patient had prior angiographic evidence of >70% stenosis in a major epicardial coronary artery or branch, history of coronary revascularization, or prior MI. Chronic kidney disease (CKD) was considered present as a binary variable if the patient had been diagnosed with stage 3, 4, or 5 CKD.

Continuous variables are presented as mean±standard deviation if normally distributed and median (25%, 75%) if nonnormally distributed. Differences in continuous variables were compared using the Student *t* test for normally distributed continuous variables and the Wilcoxon rank sum test for nonnormally distributed continuous variables. Categorical variables were compared using the χ^2^ test or a Fisher's exact test when appropriate. Data are presented in graphical form using box‐and‐whisker plots, with boxes representing medians with 25th and 75th quartiles, whiskers representing 10th and 90th percentiles, and dots representing data points that fall outside the 10th and 90th percentiles.

To correct for potential confounding of survival, models were created with the following covariates: age, sex, hypertension, diabetes mellitus, hyperlipidemia, coronary artery disease, cerebrovascular accidents, CKD, chronic obstructive pulmonary disease, and outpatient medications. The model considered use of aspirin, clopidogrel, beta‐blockers, angiotensin‐converting enzyme inhibitors or angiotensin receptor blockers, and statins. Statistically significant covariates were identified by partial *F* test for continuous covariates and by likelihood ratio tests for dichotomous covariates. The final models present adjusted percentages of survival based on the beta estimates from a multiple logistic regression. Differences were considered significant at *P*<0.05.

## Results

Two hundred and seventy‐three discharges were coded with a diagnosis of “acute myocardial infarction not present on admission” of a total of 139 410 adult discharges. Of these, 48 cases were confirmed to be STEMI on the basis of independent reviews by 2 experienced cardiologists following established criteria.^2–3^ Twenty‐seven inpatient STEMIs developed at various postoperative stages, whereas 21 patients were in the hospital being treated for a nonsurgical condition. In comparison with 227 patients with outpatient STEMIs treated at our hospital during the same period, inpatient STEMI patients were older (68 [59, 79] versus 60 [50, 70] years) and more often female, had higher rates of CKD and prior cerebrovascular events, and were more likely to be taking aspirin, dual antiplatelet therapy, a beta‐blocker, and a statin drug prior to admission (Table 1). There was a trend toward a higher rate of known coronary artery disease in inpatient STEMIs (42% versus 27%; *P*=0.06).

**Table 1.** Comparison of Inpatient and Outpatient STEMI Patients

	Inpatient STEMI (n=48)	Outpatient STEMI (n=227)	*P* Value
Age (y)	68 (59, 79)	60 (50, 70)	<0.001
Sex (% female)	50%	33%	0.046
Hypertension	77%	86%	NS
Diabetes	36%	26%	NS
CKD	29%	7%	<0.001
Sleep apnea	4%	7%	NS
COPD	13%	12%	NS
CVA/TIA	23%	11%	0.036
Known CAD	42%	27%	0.059
PAD	23%	15%	NS
Home medications			
Aspirin	63%	43%	0.013
Dual antiplatelet	21%	8%	0.007
Beta‐blocker	46%	31%	0.041
Statin	52%	33%	0.011
Peak TnI (ng/mL)	13.2 (4.9, 27.5), n=29	27.2 (10.8, 55.3), n=121	0.027
Peak TnT (ng/mL)	1.4 (0.32, 5.8), n=17	3.3 (1.6, 7.1), n=102	NS
Peak CK‐MB (ng/mL)	23.3 (11.6, 58.5)	69.1 (24.6, 125.5)	<0.001
Echocardiographic EF	55 (45, 60)	55 (42, 60)	NS
Survival to discharge	60%	96%	<0.001

UNC Hospitals switched from using troponin T (TnT) to troponin I (TnI) during the period covered by this study. Echocardiographic ejection fraction (EF) was obtained within 48 hours of STEMI. STEMI indicates ST ‐elevation myocardial infarction; CKD, chronic kidney disease; COPD, chronic obstructive pulmonary disease; CVA, cerebrovascular accident; TIA, transient ischemic attack; CAD, coronary artery disease; PAD, peripheral arterial disease; CK‐MB, MB fraction of creatine kinase; NS, not statistically significant.

The event that triggered the performance of the index ECG for inpatient STEMI was most often a change in clinical status (ie, altered mental status, hypotension, and respiratory distress; n=29, 60.4%). Less frequently, an ECG was obtained in response to patient complaints (ie, chest pain, dyspnea, and/or palpitations; n=16, 33%) or changes on telemetry (ie, tachycardia, ST‐segment deviation; n=3, 6.6%). Nine of the cases were identified by an ECG obtained after the discovery of elevated cardiac biomarkers.

**Figure 1.**
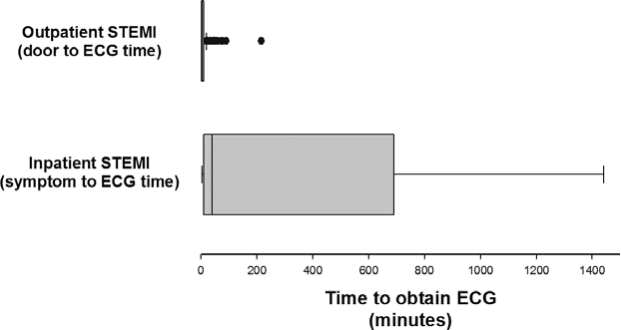
Time to obtain ECG in patients with inpatient versus outpatient STEMI. ECG indicates electrocardiogram; STEMI, ST‐elevation myocardial infarction.

The time between onset of the ischemic event and the performance of the index ECG varied dramatically, from a few minutes to more than 48 hours (Figure 1). The median time to obtain an ECG was 41 (10, 660) minutes in the 44 patients in whom the timing of the onset of symptoms could be identified. In the other 4 patients, the symptoms were either nonspecific (eg, increasing somnolence) or the nursing documentation was incomplete. By comparison, the median door to ECG time was 5 (2, 10) minutes in patients who presented to the emergency department with outpatient STEMI (*P*<0.001).

Inpatient STEMI patients who had an ECG performed within 1 hour (n=23) were more likely to have symptoms rather than a change in clinical status as the event that precipitated the ECG (61% versus 8%; *P*<0.001) and had a higher rate of revascularization (87% versus 56%; *P*=0.018) compared with patients who had an ECG performed >1 hour from the event. There was no significant difference in demographic data between these 2 groups (data not shown).

Emergent coronary angiography was performed in 71% of patients with inpatient STEMI. The other patients were deemed not to be candidates for coronary angiography because of active or excessive risk of bleeding (n=5), acute neurological symptoms and/or altered mental status (n=2), family and/or patient wishes (n=3), and comorbidities (eg, severe aortic stenosis, pulmonary embolus, ischemic bowel, CKD; n=3). One patient died awaiting transport to the cardiac catheterization laboratory.

Emergent PCI was performed in 27 patients (56% of patients with inpatient STEMI). Reasons for not performing PCI in patients who underwent coronary angiography included occlusion of a saphenous vein graft (n=1), resolution of ST elevation with intracoronary bolus of nitroglycerin (n=1), nonobstructive coronary artery disease (n=1), severe 3‐vessel coronary artery disease with either occlusion of a branch artery or no clear infarct related artery (n=3), and death prior to performance of PCI (n=1). The infarct‐related artery was the left anterior descending artery in 12 patients, the right coronary artery in 12 patients, and the left circumflex in 3 patients. Bare‐metal stents were used in 17 patients, drug‐eluting stents in 6 patients, and balloon angioplasty in 4 patients.

**Figure 2.**
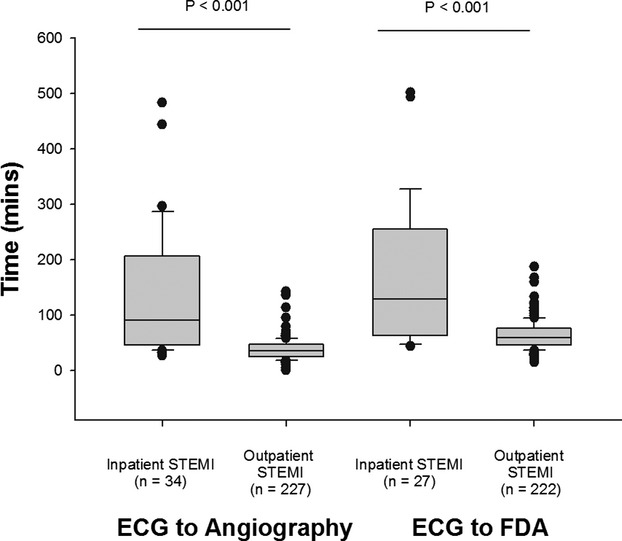
Diagnostic and treatment times in patients with STEMI. ECG‐to‐angiography and ECG‐to–first device application (FDA) times for inpatient and outpatient STEMI patients. STEMI indicates ST‐elevation myocardial infarction; ECG, electrocardiogram.

The median ECG‐to‐angiography time was 91 (26, 209) minutes for inpatient STEMI patients compared with 35 (25, 46) minutes for outpatient STEMI patients (*P*<0.001; Figure 2 and Table 2). Median ECG to first device activation (FDA) was 129 minutes in inpatient STEMI compared with 60 minutes for those with outpatient STEMI (*P*<0.001). There was no difference in time of arrival in the Cardiac Catheterization Laboratory to FDA between the 2 groups (Table 2).

**Table 2.** Time Required for Coronary Angiography and Revascularization in Patients With Inpatient or Outpatient STEMI Who Underwent Coronary Angiography

	Inpatient STEMI With Coronary Angiography (n=34)	Inpatient STEMI With PCI (n=27)	Outpatient STEMI (n=227)	Comparison of Inpatient STEMI With PCI vs Outpatient STEMI
ECG to angiography	91 (46, 223)	97 (50, 227)	35 (25, 46)	*P*<0.001
Arrival in lab to FDA		26 (15, 49)	23 (17, 30)	*P*=NS
ECG to FDA		129 (65, 250)	60 (47, 76)	*P*<0.001

STEMI indicates ST‐elevation myocardial infarction; PCI, percutaneous coronary intervention; ECG, electrocardiogram; FDA, first device activation; NS, not statistically significant.

In‐hospital mortality was 39.6% for inpatient STEMI patients compared with a rate of 4% for outpatient STEMI patients (*P*<0.001). This marked difference in mortality occurred despite peak cardiac biomarkers being higher in patients with outpatient STEMIs and postinfarct left ventricular systolic function being the same in the 2 groups (Table 1). The difference in survival persisted after adjustment for age and sex and after adjustment for age, sex, history of known coronary artery disease, prior cerebrovascular event, CKD, and use of clopidogrel (Table 3). There was a marked difference in survival comparing inpatient STEMI patients who had PCI and outpatient STEMI patients (Table 3), a difference that persisted after adjusting for potential confounders.

**Table 3.** Multivariate Analysis of the Difference in Survival Between Inpatient and Outpatient STEMI

	Unadjusted Survival		Adjusted for Age and Sex		Adjusted for Age, Sex, History of CAD, Prior CVA, CKD, and Clopidogrel Usage	
Outpatient STEMI	96.1%	<0.001	96.8%	<0.001	96.9%	<0.001
Inpatient STEMI	60.4%		70.5%		63.7%	
					Adjusted for age, sex, history of CAD	
Outpatient STEMI	96.1%	<0.001	97.2%	<0.001	97.3%	<0.001
Inpatient STEMI with PCI	67.6%		76.4%		70.3%	

Likelihood analysis was used to identify potential confounders. STEMI indicates ST‐elevation myocardial infarction; CAD, coronary artery disease; CVA, cerebrovascular accident; CKD, chronic kidney disease; PCI, percutaneous coronary intervention.

There were no statistically significant predictors of survival for inpatient STEMI patients although there were trends that patients who died were older and more likely to have known coronary artery disease and diabetes (Table 4). There was a trend toward survival being higher among patients who underwent PCI (67.6% versus 42.9%; *P*=0.11), which was further strengthened after adjusting for diabetes and known coronary artery disease (70.3% versus 38.8%; *P*=0.07). These data must be interpreted very cautiously because of the small sample size. The average hospital stay for patients who survived an inpatient STEMI was 16±29 days.

**Table 4.** Demographics, Comorbidities, and Treatment Times in Patients Who Did and Did Not Survive an Inpatient STEMI

	Survival to Discharge (n=29)	All‐Cause Morality (n=19)	*P* Value
Age (y)	66 (58, 75)	73 (66, 80)	0.08
Sex (% female)	45%	58%	0.56
Hypertension	69%	89%	0.16
Diabetes	24%	53%	0.07
Hyperlipidemia	62%	68%	0.76
Chronic kidney disease	28%	32%	1.00
Known CAD	31%	58%	0.08
COPD	7%	21%	0.20
Peak CK‐MB (ng/mL)	21.0 (12.9, 60.9)	28.6 (9.9, 55.3)	0.83
Echocardiographic EF	55 (45, 60)	53 (40, 63)	0.61
Symptom to ECG <1 hour	55%	42%	0.57
ECG to angiography	91 (45, 209)	58 (50, 197)	0.78
Coronary angiography	79%	58%	0.19
PCI	62%	47%	0.38

Although there were no statistically significant differences between groups, these results must be interpreted cautiously, as the small sample size decreases the ability of the comparison to detect a meaningful difference (α<0.80 for all comparisons). STEMI indicates ST‐elevation myocardial infarction; CAD, coronary artery disease; COPD, chronic obstructive pulmonary disease; CK‐MB, MB fraction of creatine kinase; EF, ejection fraction; ECG, electrocardiogram; PCI, percutaneous coronary intervention.

## Discussion

The current study highlights significant differences between patients who develop STEMI while hospitalized for a noncardiac condition compared with patients who had onset of STEMI while outpatients. The former group was older, more likely to be female, and had a higher rate of CKD and prior cerebrovascular events. Fewer inpatient STEMI patients received coronary angiography or PCI, and the time to reperfusion was longer than for outpatient STEMI patients. There was a large amount of variability in time from the onset of MI to performance of the ECG in hospitalized patients, and ECG‐to‐angiography times were significantly longer for inpatient STEMI than for outpatient STEMI patients. Patients with inpatient STEMI had a much higher mortality rate, which was not due to a larger amount of myocardial damage, at least as assessed by peak cardiac enzymes and post‐MI echocardiographic assessment of left ventricular systolic function.

There were 2 major sources of delay in the use of reperfusion therapy in patients with inpatient STEMI. The first was the time between the initial clinical event and ECG, which varied dramatically from a few minutes to more than 48 hours. At least part of this variability was a result of atypical presentation of STEMI in this patient population. Prior studies have shown that inpatient acute MI in postoperative patients is often associated with atypical symptoms and can be difficult to diagnose.^4–6^ In our study, the clinical event that triggered the performance of the index ECG was a change in clinical status and/or changes observed on telemetry in two thirds of patients. Patients without symptoms were less likely to receive an ECG and reperfusion therapy in an expedited manner compared with patients who verbalized complaints. For the minority of patients with chest pain (n=7), the median time between symptom onset to ECG was 10 minutes.

A second major source of delay in reperfusion therapy was longer and more variable time between ECG and coronary angiography in patients with inpatient STEMI compared with patiets with outpatient STEMI. Current guidelines recommend that patients presenting with an outpatient STEMI have an ECG within 10 minutes and FDA within 90 minutes. Using an implied maximum ECG‐to‐FDA time of 80 minutes as a performance standard, 81% of outpatient STEMI patients and 32% of inpatient STEMI patients (*P*<0.001) had times that fell within recommendations for optimal care. Although the current study was not designed to identify the reasons for these delays, our experience has shown that interpretation of the ECG is slower than optimum in many of these patients. This may in part be a result of lack of education, as efforts at training hospital personnel on the importance of rapid ECG performance and interpretation in suspected STEMIs have been concentrated on the emergency department and cardiology services. Other factors that are likely to contribute to longer ECG‐to‐angiography times in these patients are comorbidities that increase the complexity of evaluating the risks and benefits of reperfusion therapy and the need for longer discussions with patients and families regarding whether to pursue aggressive therapy.

There was no difference in time of arrival in the Cardiac Catheterization Laboratory to FDA whether a patient had the onset of STEMI while an inpatient or outpatient. This is in contrast to the marked differences in the time between MI onset and coronary angiography between the 2 groups. Stated another way, once the decision was made to pursue aggressive measures and the “STEMI system” was activated, clinical care was similar no matter where the STEMI occurred.

Little has previously been published about outcomes of inpatient STEMI, but our data are consistent with prior studies showing high mortality rates for any inpatient MI. Zymslinski et al^4^ compared outcomes of inpatient MI with those of outpatient MI in the prereperfusion era. They found significantly higher mortality for inpatient MI (66% versus 22%) and concluded that it was the result of atypical presentation, higher comorbidity, and delayed recognition. More recently, Maynard et al^6^ reported that in‐hospital mortality was much higher (27.3% versus 8.6%) for 792 patients who experienced an acute inpatient MI (9.5% who had a STEMI) compared with 6262 patients with acute MI who presented as outpatients to the Department of Veterans Affairs Health System. Patients with inpatient MI were older, more likely to have atypical symptoms, and had higher rates of renal disease, cerebrovascular disease, congestive heart failure, diabetes mellitus, chronic obstructive pulmonary disease, dementia, and cancer than patients who presented to the VA system with an outpatient MI.

Inpatient STEMIs are a major health care problem, suggesting that efforts aimed at improving care of this patient population would have a large impact. We found that the incidence of inpatient STEMI in our institution was 3.4 per 10 000 hospital discharges and was associated with an in‐hospital mortality of 40%. According to the Nationwide Inpatient Sample database of the Healthcare Cost and Utilization Project, there were 33 million in‐patient stays in 2009 in the United States,^7^ which would project to 11 000 in‐patient STEMIs and ≈4300 deaths. The problem may actually be larger than these numbers; although there are very limited data on the prevalence of acute coronary syndromes in patients hospitalized for a noncardiac condition, a large, community‐based study found that non‐STEMIs occur at a rate 3 times greater than STEMIs.^8^

There are several limitations to our study. It is a single‐center, retrospective study with a small cohort. Some patients with inpatient STEMIs may have been missed in this study because of either poor coding or sudden death prior to obtaining an ECG. There are inherent inaccuracies in identifying the onset of myocardial infarction in patients without symptoms.

## Conclusions

The development of a STEMI while hospitalized for a noncardiac condition is associated with a low use of PCI and a high mortality rate. A prospective study is needed to define whether more aggressive use of primary PCI or more rapid reperfusion therapy results in a decrease in mortality in these patients, who are older and more often female and have higher rates of CKD and prior cerebrovascular events than outpatient STEMI patients. If there is a benefit to decreasing the time between the onset of coronary ischemia and revascularization in patients with inpatient STEMI, improvement in outcomes can be achieved by programs aimed at increasing inpatient STEMI awareness, encouraging early acquisition of ECG, streamlining ECG interpretation for patients not in the emergency department, and establishing effective inpatient STEMI alert systems.

## References

[1] Krumholz HM Herrin J Miller LE Drye EE Ling SM Han LF Rapp MT Bradley EH Nallamothu BK Nsa W Bratzler DW Curtis JP Improvements in door‐to‐balloon time in the United States, 2005 to 2010. Circulation. 2011; 124:1038-1045 21859971 10.1161/CIRCULATIONAHA.111.044107 PMC3598634

[2] Thygesen K Alpert JS White HD Joint ESC/ACCF/AHA/WHF Task Force for the Redefinition of Myocardial Infarction Universal definition of myocardial infarction. J Am Coll Cardiol. 2007; 50:2173-2195 18036459 10.1016/j.jacc.2007.09.011

[3] Kushner FG Hand M Smith SC Jr King SB III Anderson JL Antman EM Bailey SR Bates ER Blankenship JC Casey DE Jr Green LA Hochman JS Jacobs AK Krumholz HM Morrison DA Ornato JP Pearle DL Peterson ED Sloan MA Whitlow PL Williams DO 2009 Focused updates: ACC/AHA guidelines for the management of patients with ST‐elevation myocardial infarction (updating the 2004 guideline and 2007 focused update) and ACC/AHA/SCAI guidelines on percutaneous coronary intervention (updating the 2005 guideline and 2007 focused update) a report of the American College of Cardiology Foundation/American Heart Association Task Force on Practice Guidelines. Circulation. 2009; 120:2271-2306 19923169 10.1161/CIRCULATIONAHA.109.192663

[4] Zymslinski RW Lackland DT Keil JE Higgins JE Increased fatality and difficult diagnosis of in‐hospital acute myocardial infarction: comparison to lower mortality and more easily recognized pre‐hospital infarction. Am Heart J. 1981; 101:586-592 7223598 10.1016/0002-8703(81)90225-8

[5] Zahn R Schiele R Seidl K Kapp T Glunz HG Jagodzinski E Voigtländer T Gottwik M Berg G Thomas H Senges J Acute myocardial infarction occurring in versus out of the hospital: patient characteristics and clinical outcome. J Am Coll Cardiol. 2000; 35:1820-1826 10841230 10.1016/s0735-1097(00)00629-x

[6] Maynard C Lowy E Rumsfeld J Sales AE Sun H Kopjar B Fleming B Jesse RL Rusch R Fihn SD The prevalence and outcomes of in‐hospital acute myocardial infarction in the Department of Veterans Affairs Health System. Arch Intern Med. 2006; 166:1410-1416 16832007 10.1001/archinte.166.13.1410

[7] Healthcare Cost and Utilization Project Facts and Figures: Statistics on Hospital‐based Care in the United States, 2009. http://www.hcup-us.ahrq.gov/reports/factsandfigures/2009/pdfs/FF_2009_section2.pdf.

[8] Yeh RW Sidney S Chandra M Sorel M Selby JV Go AS Population trends in the incidence and outcomes of acute myocardial infarction. N Engl J Med. 2010; 362:2155-2165 20558366 10.1056/NEJMoa0908610

